# Supplementary material for: WordCommentsAnalyzer: A windows software tool for qualitative research
Source: F1000Res. 2018 Sep 4;7:536. Originally published 2018 May 3. [Version 2] doi: 10.12688/f1000research.14819.2 (PMC6137411; doi:10.12688/f1000research.14819.2)
Supplement: Supplementary file 3 [file f1000research-7-17614-s0002.tgz › b2a79496-4ada-47c4-9682-a99ce867836d.zip!/Eight Iranian Health Journals/The Journal of Toloo-e-Behdasht_10.docx]

© The Journal of Toloo-e-Behdasht. This work is licensed under CC BY 4.0 (https://creativecommons.org/licenses/by/4.0/).

Volume & Issue: دوره 15 - شماره 4

Title: شناسایی ارزشهای کاربران ترافیک در شهر یزد و ارتباط آن بر رفتار ترافیکی آنها (با تمرکز برکاهش تلفات وحوادث رانندگی)

http://tbj.ssu.ac.ir/article-1-2758-fa.html

مقدمه : تصادف وسایل نقلیه و موتوری یکی از عمده‌ترین دلایل مرگ و میر در سطح جهانی هستند و بالاترین نرخ این مرگ و میر به کشورهای در حال توسعه تعلق می‌گیرد. به طور متوسط هر روزه سه هزار تصادف خودرویی منجر به مرگ در سراسر جهان اتفاق می‌افتد و مرگ‌های مرتبط با وسایل نقلیه یازدهمین عامل رایج مرگ‌ و ‌میر در کشورهای در حال توسعه هستند. این مطالعه با هدف شناسایی ارزشهای کاربران ترافیک در شهر یزد و ارتباط آن بر رفتار ترافیکی آنها انجام گرفت. روش بررسی: این پژوهش از نظر روش توصیفی-تحلیلی ، از نظر هدف کاربردی ، مقطع زمانی آن ابتدای سال 96 و جامعه آماری آن کلیه  رانندگان شهر یزد بودند که بر حسب جدول کرجسی و مورگان  384 نفر به عنوان نمونه به صورت تصادفی انتخاب شدند.  بدین صورت که شهر یزد به 5 بخش تقسیم شده و پرسش گر در هر بخش به صورت تصادفی 77 نفر را از بین رانندگان مختلف از لحاظ سن و نوع وسیله در آن بخش انتخاب نموده و پرسشنامه را بعد از دادن توضیحات لازم جهت پر کردن در اختیار آنها قرار داده است.  سوالات پرسشنامه بر اساس ارزش های کاربران ترافیک در یزد بود  براساس مطالعه مبنایی، ادبیات و تلفیق آن با نتایج به دست آمده از اطلاعات تحقیقات میدانی پژوهش حاصل شده بود. برای طبقه بندی و تجزیه و تحلیل داده ها از نرم افزارهای EXCEL و 23SPSS   استفاده شد که با دادن دو جدول خلاصه مدل (Model Summary) و جدول ضرایب رگرسیونی (ANOVA)، مقادیر همبستگی بین دو متغیر(Rو R2) و سطح معنا داری(sig) مشخص شده و نتایج به دست آمده از آنها مورد تحلیل قرار گرفت. یافته ها: متغیرهای ارزش مذهبی (289/0)، ارزش اقتصادی (282/0)، ارزش اجتماعی (250/0)، ارزش دانش (199/0)، ارزش سلامتی (159/0) و ارزش وجهه خانوادگی (146/0) به ترتیب بالاترین تاثیر را روی متغیر رفتار ترافیکی داشته اند و متغیرهای ارزش مردمی، زیبایی شناختی، لذت جویانه و قدرت، تاثیر معناداری نداشته است. نتیجه گیری: با تکیه بر ارزشهایی که در یک جامعه وجود دارد می توان به نوعی همبستگی در جهت پیش بردن و برجسته کردن ارزشهای حاکم رسید، لذا رعایت ارزشهای مذکور برابر نتایج به دست آمده با توجه به همسویی با رفتارهای ترافیکی مناسب و درست باعث رعایت کردن قوانین و مقررات راهنمایی و رانندگی شده که این امر موجب نظم بخشی به ترافیک و کاهش تصادفات و تلفات  ناشی از آن می شود.

Title: مقایسه تأثیر دو روش آموزشی پمفلت و فیلم آموزشی بر آگاهی، نگرش و عملکرد کارکنان ادارات دولتی شهر یزد در مورد عوامل خطر بیماری های قلبی ـ عروقی

http://tbj.ssu.ac.ir/article-1-2337-fa.html

بیماری قلبی- عروقی یک پاندمی و مهمترین علت مرگ و میر در سراسر جهان و ایران می باشد و سرمایه گذاری درسلامت قلب و پیشگیری از بیماری های قلبی عروقی نه تنها باعث کاهش درد ، رنج و ناتوانی میلیونها نفر و جلوگیری از اتلاف منابع و افزایش بهره وری، رشد اقتصادی و رفاه می شود لذا بر آن شدیم تا تأثیر دو روش آموزشی پمفلت و فیلم آموزشی بر آگاهی، نگرش و عملکرد کارکنان مراکز دولتی شهر یزد را در مورد  عوامل خطر بیماری های  قلبی عروقی را مورد مقایسه قرار دهیم. روش بررسی: پژوهش حاضر مطالعه ای نیمه تجربی می باشد که در سال 1390 بر روی 160 نفر از کارکنان مراکز دولتی شهر یزد(مداخله آموزشی در 79 نفر فیلم آموزشی و در 81 نفر دیگر پمفلت) انجام گرفته است. روش جمع آوری اطلاعات به صورت پرسشنامه بوده که پایایی و روایی آن مورد تایید قرار گرفت که در دو مرحله قبل از آموزش و دو ماه پس از تحویل فیلم های آموزشی و پمفلت، داده ها جمع آوری و پس از وارد کردن داده ها در نرم افزار آماری SPSS با استفاده از آزمون های آماری، T-test،  Chi squareو همبستگی پیرسون مورد تجزیه و تحلیل قرار گرفت. یافته ها: بر اساس نتایج این مطالعه اختلاف میانگین نمره آگاهی، نگرش و عملکرد قبل از آموزش در هر دو گروه آموزشی اختلاف معنی داری نداشته(05/0P >)ولی بعد از آموزش اختلاف معنی دار آماری بین میانگین نمره نگرش و عملکرد بر اساس پمفلت و فیلم آموزشی از لحاظ آماری معنی دار بوده(01/0P <) بطوری که تاثیر فیلم آموزشی بیشتر از پمفلت بود. بطور کلی هر دو روش آموزشی بر آگاهی، نگرش و عملکرد کارکنان موثر بود(01/0P <). بحث و نتیجه گیری: با توجه به اینکه هر دو روش آموزشی فیلم آموزشی و پمفلت بر آگاهی، نگرش و عملکرد کارکنان تاثیر داشته هر چند تاثیر فیلم آموزشی بر نگرش و عملکرد آنها بیشتر بود، می توان نتیجه گیری کرد که هر دو می توانند روش موثری بر ارتقای سطح آگاهی، نگرش و عملکرد در مورد عوامل خطر بیماری های قلبی و عروقی باشد.

Title: حذف سرب از محلول آبی سنتتیک بوسیله خاکستر پسماند مواد غذایی

http://tbj.ssu.ac.ir/article-1-2338-fa.html

چکیده مقدمه:سرب از جمله فلزات سنگینسمی در برخی از فاضلاب های صنعتی بوده که می‌تواند اثرات نامطلوب بر محیط و سلامت انسان به همراه داشته باشد. از تکنولوژی‌های امید بخش برای حذف آلاینده ها از آب، استفاده از فرآیند جذب سطحی است. در این مطالعه‌، تاثیر خاکستر پسماند مواد غذایی به عنوان یک جاذب ارزان قیمت، سازگار با محیط زیست و در دسترس، در حذف سرب از محلول‌های آبی سنتتیک مورد بررسی قرار گرفته است. روش بررسی: در مطالعه حاضر که یک مطالعه تجربی است حذف سرب بوسیله جاذب خاکستر پسماند مواد غذایی در سیستم ناپیوسته مورد بررسی قرار گرفت. اثر پارامتر های مختلف موثر  در جذب از جمله دز جاذب، زمان تماس، pH ، غلظت اولیه سرب و دما نیز در فرایند جذب مطالعه گردید. به منظور تحلیل نتایج از نرم افزار اکسل استفاده شده و سپس رفتار فرآیند جذب با مدل های ایزوترمی لانگمویر و فروندلیچ مدل سازی شد. یافته ها: مطالعه نشان داد که افزایش غلظت اولیه سرب راندمان حذف را کاهش می دهد. راندمان حذف سرب بوسیله خاکستر پسماند مواد غذایی با افزایش pH و دما، افزایش می یابد. همچنین با بررسی نتایج بدست آمده مشخص شد ظرفیت جذب خاکستر پسماند مواد غذایی نسبت به بسیاری از جاذب های مطالعه شده بیشتر بوده و فرآیند بطور قابل قبولی از ایزوترم فروندلیچ تبعیت می کند. نتیجه گیری: نتایج حاصل از این مطالعه استفاده از جاذب خاکستر پسماند مواد غذایی را با توجه به ارزان و دردسترس بودن و نیز ظرفیت بالای آن در جذب سرب، در سیستم های جذب واقعی تایید می نماید.

Title: بررسی اثر آنتی باکتریالی عصاره هیدروالکلی نعناع فلفلی بر شش باکتری بیماری زا در مواد غذایی

http://tbj.ssu.ac.ir/article-1-2339-fa.html

چکیده مقدمه: کشور ایران از نظر پراکندگی و گستردگی گیاهان دارویی از غنی ترین مناطق جهان محسوب می شود. امروزه اثر ضد میکروبی اسانس و عصاره های گیاهی بخوبی مشخص شده و به عنوان جایگزینی مناسب، در صنعت غذا جهت کنترل پاتوژنهای غذا زاد استفاده می گردد.با توجه به فعالیت ضد باکتریایی عصاره ها و کارایی آنها علیه میکروارگانیسم ها هدف از انجام این تحقیق بررسی فعالیت ضد باکتریایی عصاره نعناع فلفلی به منظور کنترل باکتری های بیماریزا بود. روش بررسی: در این مطالعه تجربی- آزمایشگاهی از گونه Piperita L. ، یکی از گونه های نعناع استفاده شد و عصاره گیری به روش پرکولاتور انجام گردید. اثر ضد میکروبی عصاره با استفاده از روش  چاهک- آگار، حداقل غلظت مهار کنندگی (MIC) و حداقل غلظت کشندگی (MBC)  با روش میکرودایلوشن بر روی شش باکتری استاندارد انجام شد. آزمون برای هر کدام از باکتری ها با 3 تکرار انجام  پذیرفت و داده های جمع آوری شده با استفاده از نرم افزار SPSS نسخه 16 و آزمون تی مورد تجزیه و تحلیل قرار گرفت. یافته ها: در این پژوهش کمترین غلظت ممانعت کنندگی عصاره نعناع بر میکروارگانیسم های مورد آزمایش برابر با 25/3 میلی گرم بر میلی لیتر و در مورد باکتری های Staphylococcus aureus و Enterococcus faecalis مشاهده شد. همچنین بیشترین قطر هاله عدم رشد مربوط به باکتری Staphylococcus aureus  (32 میلی متر) بود. نتیجه گیری: نتایج این مطالعه نشان داد که عصاره نعناع فلفلی اثر کنترل کنندگی مناسبی در رشد پاتوژنهای منتقله از مواد غذایی دارد که می توان از آن بعنوان یک نگهدارنده مناسب جهت حفظ و نگهداری مواد غذایی استفاده نمود.  این مقاله حاصل پایان نامه دانشجویی رشته کارشناسی ارشد بهداشت و ایمنی مواد غذایی دانشکده بهداشت دانشگاه علوم پزشکی شهید صدوقی یزد می باشد.

Title: تحلیل عوامل موثر بر تشخیص رتینوپاتی در بیماران مبتلا به دیابت نوع2 با استفاده از مدل‌های پارامتری و مدل کاکس

http://tbj.ssu.ac.ir/article-1-2343-fa.html

چکیده مقدمه: یکی از مهم‌ترین عوارض دیابت ، رتینوپاتی دیابتی است که یکی از علل عمده از دست دادن بینایی بشمار می‌رود و سالانه باعث کوری بیش از 10000 نفر می‌گردد.هدف این مطالعه تعیین عوامل موثر بر تشخیص رتینوپاتی در بیماران مبتلا به دیابت نوع 2 می‌باشد.  روش بررسی:در این پژوهش آینده نگر تاریخی ، 400 بیمار مبتلا به دیابت نوع 2 و فاقد رتینوپاتی که در سال 1387 به کلینیک چشم پزشکی مرکز تحقیقات دیابت یزد مراجعه نموده‌اند، وارد مطالعه شده‌اند و تا بهمن ماه 1391 از نظر تشخیص رتینوپاتی پیگیری شدند. برای شناسایی عوامل موثر بر تشخیص رتینوپاتی از مدل نیمه پارامتری کاکس و مدل‌های پارامتری: نمایی ، وایبل ، لگ نرمال و لگ لجستیک استفاده شده است. یافته‌ها: مصرف آسپرین 6/0برابر (در مدل وایبل ، لگ نرمال) و یک برابر (در مدل کاکس) خطر تشخیص رتینوپاتی را افزایش می‌دهد. به عبارتی سبب کاهش زمان تشخیص رتینوپاتی در این افراد می‌شود. در مطالعه حاضر خطر تشخیص رتینوپاتی دیابتی در بیمارانی که آنمی داشته‌اند، 4/1 برابر بیمارانی است، که فاقد این عامل خطر بوده‌اند. داشتن آنمی موجب تشخیص زودتر رتینوپاتی دیابتی می‌گردد( 05/0P <). مصرف انسولین باعث تا خیر در زمان تشخیص رتینوپاتی می‌شود و خطر تشخیص رتینوپاتی دیابتی را کاهش می‌دهد. نتیجه گیری: کنترل قند خون ، معاینه منظم افراد دیابتی توسط متخصصان چشم پزشکی در مراحل اولیه تشخیص دیابت، کنترل مصرف آسپرین ،تشخیص و درمان آنمی، در بیماران مبتلا به دیابت نوع 2 پیشنهاد می‌شود.  این مقاله حاصل پایان نامه پایان نامه کارشناسی ارشد رشته آمار زیستی دانشگاه شهید صدوقی یزد می باشد.

Title: بررسی رابطه بین سبک مدیریت مربیگرا و چابکی سازمان در دانشگاه علوم پزشکی شهید صدوقی یزد در سال 1393

http://tbj.ssu.ac.ir/article-1-2346-fa.html

چکیده مقدمه: نظام آموزش عالی و دانشگاه ها جهت بقا در فضای تنوع و پیچیدگی نیازمند تغییر و یادگیری هستند. لذا هدف این تحقیق بررسی رابطه بین سبک مدیریت مربیگرا به عنوان یک روش جدید مدیریتی با زبان یادگیری ضمنی و چابکی سازمانی به عنوان یک توانایی  تغییر مداوم و غیرقابل پیش بینی است. روش بررسی: این تحقیق از نوع هدف کاربردی  و از حیث ماهیت توصیفی- همبستگی است؛ همچنین از آنجا که با استفاده از پرسشنامه های استاندارد مدیریت مربیگری مک لین و همکاران و چابکی سازمانی شریفی و ژانگ با مقیاس رتبه ای لیکرت به صورت میدانی به جمع آوری داده ها پرداخته شده است، یک تحقیق پیمایشی است. آلفای کرونباخ به دست آمده از 218 پرسشنامه جمع آوری شده از بین کارکنان دانشگاه علوم پزشکی که با فرمول کوکران به دست آمد، برای سبک مدیریت مربیگرا 82/0 و چابکی 95/0 است. یافته ها: نتایج نشان داد که شیوه مدیریت مربیگری در دانشگاه علوم پزشکی با بعد پاسخگویی و شایستگی در حد متوسط و با ابعاد انعطاف­پذیری و سرعت در حد ضعیف رابطه داشته است. همچنین آمار توصیفی حاکی از آن است که مدیریت مربیگرا و چابکی در حد متوسط در دانشگاه علوم پزشکی شهید صدوقی وجود دارند و بین این دو متغیر با 258/0R=  و با 95/0 درصد اطمینان رابطه وجود دارد. تمام تجزیه و تحلیل داده ها با استفاده از نرم افزار  spss ویراست 20 انجام شده است. نتیجه گیری: نتایج تحقیق نشان دهنده رابطه مثبت ومعنادار بین سبک مدیریت مربیگرا و چابکی سازمانی است. لذا سازمانهایی که به دنبال مدیریت مربیگرا و انجام کارهای تیمی هستند باید بدانند که پاسخگویی مناسب ارزش آفرین، شایستگی باعث توسعه و پیشرفت دانش و مهارت در بین افراد، انعطاف پذیری، بالا بودن کیفیت تولیدات و وجود نوآوری های جدید و وجود سرعت بیشتر در مقابل تغییرات و تحولات سازمان را به همراه خواهد داشت.

Title: متاژنومیکس و کاربرد‌های آن

http://tbj.ssu.ac.ir/article-1-2345-fa.html

مقدمه: باکتریها گروهی از میکروارگانیسم­ها هستند که در مقایسه با تنوع بالای آنها در طبیعت، تنها تعداد بسیار اندکی از آنها در محیطهای آزمایشگاهی استاندارد، قابل رشد و جدا سازی می­باشند. متاژنومیکس به عنوان یک رشته جدید پژوهشی است، که در طول دهه گذشته به بررسی ژنومهای میکروبی غیر قابل کشت می­پردازد. محققان در مناطق مختلف جهان با مطالعه جدی این گروه از باکتریها، بدنبال یافتن ترکیباتی همچون آنتی­بیوتیک­های جدید، ترکیبات ضد سرطان، آنزیم­های جدید و مولکولهای زیستی هستند. روش بررسی: این مقاله یک مطالعه مروری است که با بررسی متون و جستجوی اینترنتی و دستی کلید‌ واژه‌های متاژنومیکس،  بیوتکنولوژی، محیط زیست، میکرو ارگانیسم و   آنزیم از  منابع و            سایت­های معتبر علمی ازجمله Google scholar ، Pub med، Science direct، Sid و Scopus در بین سالهای 2000 تا 2013جمع­آوری و مورد مطالعه قرار گرفت. ابزار گردآوری اطلاعات در این پژوهش شامل همه متون چاپ شده در زمینه متاژنومیکس است. یافته‌ها: اگرچه متانوژنومیک امروزه برای غربالگری استفاده می­شود اما در حال حاضر به عنوان یک تکنیک کامل در کنار کاربرد محیط کشت و دیگر فنون سنتی موقعیت بهتری پیدا خواهد کرد. بیشترین میزان کاربرد متانوژنومیک در موارد بالینی در جایی است که با تکنیک­های متداول، نمی­توان دلایل میکروبی را کشف کرد بنابراین برای انجام آزمایشات و آنالیز اطلاعات به دانشمندان ماهر نیاز است. نتیجه­گیری: این مقاله مروری بر برخی از جدیدترین دستاوردهای حاصل از متاژنومیکس تکیه دارد و کاربرد آن­را در مواد دارویی جدید، کشف آنزیم­ها، پتانسیل بیوتکنولوژی و محیط زیست بررسی می­کند.

Title: رفتارهای پیشگیری کننده از عفونت واژینال-کاندیدا آلبیکنس در زنان دیابتی بر اساس مدل اعتقاد بهداشتی در شهر یزد

http://tbj.ssu.ac.ir/article-1-2348-fa.html

چکیده مقدمه:کاندیدیازیس یک معضل جهانی است که میلیون ها زن را در هر سال درگیر می کند.کاندیدا آلبیکنس عامل 90-85 درصد از عفونت های قارچی واژن شناخته شده است. هدف از مطالعه حاضر تعیین رفتارهای پیشگیری کننده از عفونت های واژینال-کاندیدا آلبیکنس در زنان دیابتی بر اساس مدل اعتقاد بهداشتی بوده است. تا کنون در این زمینه تحقیقی با این عنوان انجام نشده است. روش بررسی:مطالعه حاضربه صورت توصیفی- تحلیلی مقطعی می باشدکه بر روی نمونه متشکل از 300 زن مراجعه کننده به کلینیک مرکز دیابت شهر یزدومطب پزشکان فوق تخصص غدد بوده است که به صورت تصادفی ساده  300 پرونده انتخاب و برای شرکت در مطالعه با کسب رضایت از آنها دعوت به عمل آمد. ابزار گردآوری داده ها پرسشنامه محقق ساخته ای بوده که روایی و  پایایی آن مورد تایید قرار گرفت.داده ها با  استفاده از آزمون های آماری پارامتریک نرم افزار آماری spssنسخه 16 تجزیه و تحلیل شد. یافته ها:میانگین سنی نمونه­ها 84/6±1/50 سال بود.همچنین میانگین نمره آگاهی آنهااز عفونت کاندیدا آلبیکنس واژینال 69/2±19/18(دامنه نمرات 21-0)، حساسیت درک شده نسبت به عفونت کاندیدا آلبیکنس واژینال 70/2±50/5 (دامنه نمرات 8-0)، شدت درک شده80/2±99/6(دامنه نمرات 12-0)، منافع درک شده 60/2±80/19(دامنه نمرات 22-0)، موانع درک شده  65/2±05/4(دامنه نمرات 14-0)، راهنما برای عمل80/1±34/2(دامنه نمرات 7-0)، خودکارآمدی 52/1±12/11(دامنه نمرات 12-0) بوده است. سازه های مدل توانسته اند 242/0از واریانس رفتارهای پیشگیری کننده از عفونت کاندیدا آلبیکنس واژینال را پیش بینی کنند که در این میان  خودکارآمدی  قویترین سازه بوده است. بحث و نتیجه گیری:با توجه به نتایج مطالعه حاضر که سطح آگاهی و رفتار زنان مراجعه کننده به کلینیک مرکز دیابت در سطح نسبتاً مناسبی قرار داشت.پیشنهاد می شود تا ادامه و تقویت برنامه های آموزشی مبتنی بر الگوی اعتقاد بهداشتی و با تاکید بر خودکارآمدی به منظور بهبود رفتارهای پیشگیری کننده از عفونت کاندیدا آلبیکنس واژینال صورت گیرد. این مقاله حاصل پایان نامه کارشناسی ارشد آموزش بهداشت دانشگاه علوم پزشکی و خدمات بهداشتی درمانی شهید صدوقی یزد می باشد.

Title: بررسی خصوصیات فیزیکوشیمیایی و میکروبی فیلم‌های تهیه شده حاوی نانوذرات اکسید تیتانیوم با پایه پلی ساکارید آرد سویا

http://tbj.ssu.ac.ir/article-1-2349-fa.html

مقدمه: بیوپلیمرهای مشتق شده از منابع طبیعی که سهم زیادی از توجهات را در سال های اخیر بخود جلب نموده اند، بعنوان جایگزین های فیلم های پلاستیکی غیرزیست تخریب پذیر متداول در نظر گرفته می شوند چرا که هزینه آن ها پایین است و به آسانی از منابع تجدیدپذیر و زیست تخریب پذیر در دسترس اند. در این پژوهش، تاثیر غلظتهای مختلف اکسید تیتانیوم بر ویژگی های فیزیکوشیمیایی و میکروبی فیلم خوراکی بر پایه پلی ساکارید محلول دانه سویا بررسی گردید. روش بررسی: فیلم­های نانوکامپوزیتی با افزودن نانو ذرات اکسید تیتانیوم در غلظت­های مختلف (5، 10 و 15 درصد بر پایه وزن خشک) به پلی ساکارید آرد سویا، تولید گردید. نانو کامپوزیت حاصل، با هدف بررسی ویژگی­های فیزیکوشیمیایی و میکروبی به روش قالب­ریزی محلول، سنتز شد. یافته ها: با افزایش غلظت نانوذرات در فیلم ها، میزان رطوبت و حلالیت آنها به طور معنی داری کاهش یافت اما میزان مقاومت کششی آنها به طور معنی داری افزایش یافت. نانواکسید تیتانیوم دارای اثر بازدارندگی زیادی بر روی سه باکتری باسیلوس سرئوس واستافیلوکوکوس اورئوس و استافیلوکوکوس اپیدرمیدیس بود. فیلم های تولید شده، اثری بر روی حداقل غلظت بازدارندگی و کشندگی کپک ها نداشتند. با افزایش غلظت نانوذره اکسید تیتانیوم، درصدمهارکنندگی آن بر کپک پنیسیلیوم اکسپانسوم افزایش معنی داری نشان داد. نتیجه گیری: نتایج نشان داد که نانوذرات اکسید تیتانیوم می توانند در ماتریس پروتئینی سویا برای تولید فیلم استفاده گردند. فیلم کامپوزیتی تولید شده در این پژوهش را می توان برای کاربردهای غذایی و به عنوان ماده بسته بندی زیست تخریب پذیر استفاده نمود.

Title: بررسی کارایی نانو ذرات آهن اکسید سنتز شده در حذف یون مس از محلول‌های آبی

http://tbj.ssu.ac.ir/article-1-2351-fa.html

چکیده مقدمه: آلودگی منابع آب درنتیجه‌ی ورود مستقیم یون‌های فلزی از پساب‌های صنعتی، یکی از دغدغه‌های مهم جهان امروز است. در این مطالعه حذف یون مس از آب‌های آلوده به‌وسیله نانو ذرات آهن اکسید سنتز شده در حضور عصاره پوست نارنگی موردبررسی قرار گرفت. روش بررسی: برای تهیه نانو ذرات آهن اکسید از روش هم رسوبی استفاده شد. برای جلوگیری از تجمع و کاهش قطر ذرات از عصاره پوست نارنگی استفاده شد. اثر پارامترهای مختلف مانند زمان تماس، pH، غلظت اولیه یون مس و مقدار جاذب بر کارایی فرایند حذف آلاینده بررسی و بهینه گردید. جهت مطالعه بیشتر فرایند جذب، داده­های آزمایش با مدل­های جذب لانگمویر و فروندلیچ  برازش و بررسی شدند. یافته‌ها: نتایج نشان داند، درصد حذف یون مس با افزایش pH محلول، افزایش‌یافته است. افزایش غلظت یون مس از 5 به 10 میلی‌گرم در لیتر، درصد حذف آن را از 88 درصد به 81 درصد کاهش یافت. بیشترین درصد حذف یون مس، هنگامی­که مقدار جاذب 6/0 گرم و غلظت آلاینده 5 میلی‌گرم در لیتر بود، به 92% رسید. نتیجه‌گیری: نتایج آزمایش­های جذب مس نشان داد که فرایند حذف یون مس به‌طور معنی‌داری وابسته به pH است و درصد حذف رابطه معکوس با غلظت یون مس در محلول دارد. فرایند جذب این آلاینده به‌وسیله نانو ذرات آهن اکسید از مدل ایزوترم لانگمویر پیروی می­کند. نتایج این پژوهش نشان داد که نانو ذرات آهن اکسید سنتز شده در حضور عصاره پوست نارنگی برای حذف فلزات سنگین از محلول‌های آبی، مناسب و کارآمد می­باشند.

Title: بررسی تاثیرآموزش سبک های مقابله بااسترس برکاهش سطح اضطراب واسترس دانش آموزان دخترهنرستان های شهرستان نیریز

http://tbj.ssu.ac.ir/article-1-2352-fa.html

مقدمه:اضطراب و استرس ناشی از تنش، جزء جدایی ناپذیرتجربه و طبیعت بشر محسوب میشود که علاوه بر اثرات مثبت می تواند اثرات سویی بر زندگی فرد داشته باشد. هدف از انجام این مطالعه بررسی تاثیرآموزش سبک های مقابله بااسترس برکاهش سطح اضطراب واسترس دانش اموزان می باشد. روش بررسی:این مطالعه به صورت نیمه تجربی از نوع پیش آزمون  پس آزمون و بر روی 280 دانش آموز دختر هنرستانی انجام شد. که به دو گروه مورد و شاهد تقسیم شده و به گروه مورد آموزش داده شد.ابزار جمع­آوری اطلاعات دو پرسشنامه شامل، پرسشنامه 14 سوالی استرس ادراک شده(PSS) و پرسشنامه اضطراب کتل می بود. یافته ها:میانگین اضطراب قبل و بعد از آموزش به ترتیب 02/10± 17/35 و 02/11± 98/26 و استرس 39/7± 13/29 و 86/10±67/25 می بود.در مقایسه شغل پدر و میزان اضطراب و استرس هیچ رابطه معنی داری میان این متغیرها یافت نشد(217/0=P و 59/0=P). همچنین میزان تحصیلات والیدین نیز هیچ اثری بر میزان استرس و اضراب نداشت(464/=P و 885/0=P  ). شدت استرس و اضطراب بعد از آموزش کاهش معنی داری داشت(000/0=P). نتیجه گیری:با توجه به نتایج به دست آمده می توان گفت آموزش روش های مقابله با استرس و اضطراب می تواند از میزان آن ها کاسته و از این طریق بر تاثیر مثبت بر سلامت روان و تحصیل دانش آموزان داشته باشد.

Title: بررسی قابلیت حذف پیش سازهای تری هالومتان توسط ترکیبات Al+3 و Fe +3 از آب رودخانه شهرچای ارومیه

http://tbj.ssu.ac.ir/article-1-2353-fa.html

مقدمه: در بین محصولاتِ جانبی ناشی از کلرزنی آب، تری­هالومتان (THMs)  به عنوان محصول سرطانزایِ عمده شناخته شده است که پیش ساز اصلی آن ترکیبات هیومیکی می باشد. هدف این مطالعه بررسی قابلیت حذف پیش­سازهای تری­هالومتان توسط ترکیبات  Al+3 و  Fe +3 از آب رودخانه شهرچای ارومیه بوده است. روش  بررسی: این مطالعه به صورت تجربی انجام شده است. آزمایشات در راکتور ناپیوسته جار ِتست در مقادیر مختلف pH ، Al+3 و  Fe +3 انجام گرفت مقادیر THMs هم تحت شرایط بهینه حذف اندازه گیری شد. یافته ها: دوز بهینه کلروفریک، الوم و PACl برای حذف ترکیبات هیومیکی به ترتیب10، 30 و mg/L20 به دست آمد که کلروفریک با راندمان حذف 89 درصد بالاترین کارایی را داشته است و PACl با 88 درصد و الوم با 81 درصد در رتبه بعدی قرار داشتند ولی مقادیر شاخص جذب ویژه اشعه ماورای بنفش فقط برای کلروفریک کمتر از محدوده مجاز USEPA (کمتر ازL/mg m  2) به دست آمد. همچنین در شرایط بهینه مقادیر pH و تری هالومتان به ترتیب بالای 7 و کمتر از ppb50 حاصل شد نتیجه گیری: کلروفریک می تواند برای حذف اسید هیومیک رودخانه شهرچای ارومیه مورد استفاده قرار گیرد و شاخص جذب ویژه اشعه ماورای بنفش را به محدوده مجاز کاهش دهد ولی کاربرد نمک های الومینیوم امکان تشکیل THMs را افزایش می دهد. کلرزنی آب تصفیه شده با کلروفریک کاهش مقادیر تری هالومتان به محدوده مجاز USEPA (ppb<80 ) را تایید نمود.

Title: بررسی آگاهی،نگرش و عملکرد مسافرین عازم به خارج از کشور در خصوص پیشگیری از بیماری های ایدز، هپاتیت B و انفلوانزا

http://tbj.ssu.ac.ir/article-1-795-fa.html

مقدمه: سالانه چندین میلیون نفر از ایرانیان به خارج از کشور مسافرت می نمایند.مسافران بین المللی یکی از گروههای در معرض خطر می باشند اینکه شخص به کجا مسافرت می کند چندان اهمیتی ندارد. بلکه مهم آن است که مسافران عازم به خارج ازکشور اطلاعاتی در باره راههای سرایت و پیشگیری از بیماریهای عفونی را داشته باشند و نهایتا" با دانستن آن در طول سفر، خود را از ابتلا به این بیماریهای مهلک محفوظ نگهدارند. لذا این بررسی با هدف  تعیین سطح آگاهی، نگرش و عملکرد مسافران عازم به خارج از کشور در خصوص پیشگیری از بیماری های ایدز، هپاتیت B و انفلوانزا  انجام شده است. روش بررسی: این مطالعه توصیفی از نوع مقطعی با حجم نمونه 207 مسافر عازم  به خارج از کشور در فرودگاه امام خمینی انجام گردید. نمونه گیری بصورت تصادفی ساده وروش جمع آوری  اطلاعات از طریق پرسشنامه بوده که پایایی وروایی آن مورد تایید قرار گرفت . داده ها  از طریق جداول توزیع فراوانی، آزمون های آماری Anova،  T testو  cross tab درنرم افزار SPSS-16 تجزیه و تحلیل گردید. یافته ها: از 207 مسافر مورد بررسی بیشتر افراد متاهل (2/60%) بودند. میانگین سنی آنها 31/10 ± 66/34 بود و سطح تحصیلات 3/47%از آنها فوق دیپلم و لیسانس بود. بالاترین میزان آگاهی مسافران نسبت به بیماری ایدز با  5/81%، آنفلوآنزا 80%، در حالیکه نسبت به بیماری هپاتیتB  آگاهی کمتری(5/48%) داشتند. در هر سه بیماری افراد دارای نگرشی خوب ومثبتی در خصوص پیشگیری  داشتند. همچنین بالاترین عملکرد مسافران هم مربوط به بیماری ایدز بود.  نتیجه گیری: با توجه به نتایج این پژوهش مبنی بر آگاهی و عملکرد متوسط و نگرش خوب مسافران و با توجه به این که مسافرت های بین المللی می تواند موجب انتقال واپیدمی بیماریها شود، لذا پیشنهاد می گردد جهت آموزش مسافران به خارج از کشور از طریق برنامه مدون آموزشی، پوستر، پمفلت، رادیو وتلویزیون اقدامات عاجلی انجام گردد.

Title: بررسی شیوع کم خونی فقرآهن در زنان باردار دریافت کننده ی خدمات آهن یاری شهر اردکان در مقاطع سه ماهه ی اول و سه ماهه ی ‏سوم بارداری و نحوه ی دریافت خدمات آهن یاری در آن گروه

http://tbj.ssu.ac.ir/article-1-1541-fa.html

چکیده و هدف:‏ کم خونی ناشی از فقر آهن یکی از مسایل بهداشتی مهم در بین زنان باردار است که موجب زایمان زودرس، وزن کم هنگام تولد یا تولد نوزاد مرده و... ‏می گردد. هدف از این مطالعه بررسی شیوع کم خونی فقرآهن در زنان باردار دریافت کننده ی خدمات آهن یاری شهر اردکان در مقاطع سه ماهه ی اول ‏و سه ماهه ی سوم بارداری و نحوه ی دریافت خدمات آهن یاری در آن گروه در زمستان 1390 بود.‏ روش بررسی: پژوهش حاضر، یک مطالعه توصیفی، تحلیلی، و از نوع مقطعی می باشد. جامعه آماری این پژوهش، شامل زنان باردار دارای پرونده در مراکز ‏بهداشتی درمانی شهری اردکان می باشد.‏ حجم نمونه 393 زن باردار تعیین گردید. روش نمونه گیری به صورت آسان از بین مادران باردار مراجعه کننده به مراکز بهداشتی درمانی شهری اردکان ‏انتخاب شد. در این پژوهش ابزار گردآوری داده ها پرسشنامه محقق ساخت حاوی 11سؤال در7 مولفه بود که در دو مقطع سه ماهه ی اول وسوم به ‏صورت مجزا تکمیل و نتایج آن استخراج ومحاسبه گردید و با توجه به آزمونهای آماری توصیفی و کی- دو در سطح معنی داری(01/0 ‏p<‎‏ ) انجام و مورد ‏آنالیز وتحلیل قرارگرفت.‏ یافته ها: نتایج مطالعه حاضر نشان می دهد که فراوانی کم خونی در سه ماهه سوم بارداری( با میانگین هموگلوبین 34/11 )، ( 4/36% )بوده، درحالیکه همین ‏فراوانی در سه ماهه اول بارداری( با میانگین هموگلوبین33/12) (9/9%)می باشد که روند افزایشی میزان کم خونی در زنان باردار در سه ماهه سوم را بطور ‏معنی داری‎(p<‎‏0.001‏‎)‎‏ نشان می دهد. نتایج مطالعه حاضر در مؤلفه تحصیلات ، رتبه بارداری، سن، نوع قرص آهن مصرفی، رابطه مواد مصرفی به همراه ‏مکمل آهن، و شغل زنان باردار و ارتباط آن با کم خونی ارتباط معنی داری مشاهده نشد. ‏ از یافته های دیگر زمان شروع مصرف مکمل آهن و رابطه غذاهای مصرفی به همراه مکمل آهن بود که بین این مولفه ها و کم خونی بر اساس آزمون ‏آماری کی –دو تفاوت معنی دار مشاهده گردید. همچنین زمان مصرف بر حسب اوقات شبانه روز که بر اساس آزمون آماری کی –دو تفاوت معنی داری ‏مشاهده نشد.‏

Title: بررسی علل زیربنایی وفرهنگی همبودی اختلال افسردگی عمده و وسواس فکری در بیماران مراجعه کننده به کلینیک و مراکز درمانی روانشناسی وروانپزشکی شهر تهران

http://tbj.ssu.ac.ir/article-1-1395-fa.html

چکیده مقدمه: تشخیص یک بیماری از بیماری دیگر  برای بیمار و گروه درمانگر بسیار مهم است و این امر در فرآیند تشخیص اتفاق می افتد. وقتی بیماری بیش از یک اختلال تشخیص آماری اختلال روانپزشکی دارد با تشخیص چند گانه یا همبودی مواجه است. بنابراین هدف این پژوهش بررسی  عوامل زیر بنایی مشترک در اختلال همبودی وسواس فکری و افسردگی عمده و نقش عوامل فرهنگی تجلی ساز در بروز عوامل زیربنایی  این اختلال می باشد. روش بررسی:  300 بیمار به روش نمونه گیری هدف دار از میان بیماران مراکز درمانی روانشناختی تهران انتخاب شدند، و به پرسشنامه محقق ساخته علل فرهنگی همبودی اختلال وسواس فکری و افسردگی عمده، و آزمون منش و خلق و خو کلینجر پاسخ دادند. نتایج به وسیله رگرسیون لجستیک تک متغیره و چند متغیره تجزیه و تحلیل شدند. یافته ها: نتایج نشان داد متغیر باورهای غلط همراه عدم انعطاف پذیری، کمال جویی مفرط والدین و اهمیت افراطی به نظافت و تمیزی، بدون بررسی تعامل این متغیرها با متغیرهای دیگر، در بروز همبودی این اختلال تأثیر دارند. در بخش عوامل فرهنگی ابعاد ( اهمیت افراطی به نظافت و تمیزی) و (انعطاف ناپذیری و خشونت والدین)، در بخش منش و سرشت، پشتکار، کمال جویی والدین و در بخش کل متغیرها، به وسیله مدل رگرسیون پیش بینی شدند. بحث و نتیجه گیری: این مطالعه مشخص کرد که در بروز همبودی اختلال وسواس فکری و افسردگی عمده دو عامل فرهنگی، اهمیت افراطی و نظافت و تمیزی و کمال جویی والدین در تعامل با یک زمینه ساز پشتکار منجر به بروز همبودی این اختلال می گردد.

Title: بررسی میزان مواجهه با ارتعاش تمام بدن در رانندگان تاکسی شهر یزد

http://tbj.ssu.ac.ir/article-1-1272-fa.html

چکیده مقدمه: یکی از منابع رایج ارتعاش تمام بدن ، وسایل حمل و نقل هستند که در آن ‌راننده در معرض ارتعاشهای ناشی از وسیله نقلیه و جاده قرار دارد. از جمله افرادی که پیوسته با ارتعاش تمام بدن مواجهه دارند می توان به رانندگان تاکسی اشاره کرد. رانندگان تاکسی در طول شیفت کاری خود با عوامل زیان آور متعددی مانند صدا، ارتعاش، آلودگی هوا، شیفتهای کاری طولانی و استرسهای روانی مواجه هستند. مواجهه طولانی مدت با ارتعاش تمام بدن در رانندگان تاکسی می تواند با اثرات نامطلوبی بر روی سلامت آن ها  در ارتباط باشد. روش بررسی: در این مطالعه به منظور بررسی میزان مواجهه با ارتعاش تمام بدن در رانندگان تاکسی، اندازه گیری ارتعاش در 80 تاکسی از 3 نوع خودرو (سمند، پژو405 و پراید)که از لحاظ کارکرد در 3 گروه قرار گرفتند، شامل 63 نفر راننده مرد و17 نفر راننده زن صورت گرفت. پارامترهای مربوط به ارتعاش از جمله شتاب موثر، شتاب معادل کلی بدن و همچنین دوز ارتعاش در 3 محور ثبت گردید. سپس نتایج به دست آمده با مقادیر توصیه شده توسط استاندارد (ISO 2631-1)مقایسه گردید. هدف از این مطالعه بررسی توصیفی - مقطعی مواجهه رانندگان تاکسی شهر یزد با ارتعاش تمام بدن بوده است. یافته ها: میانگین شتاب توزین یافته فرکانسی ریشه مجموع مربعات (rms)در  محور  Zبرابر با2m/s45/0 بود که از میانگین در محورهای X , Y بیشتر بود. اکثریت مقادیر شتاب موثر(rms) در محدوده خطرات بالقوه بهداشتی قرار داشت. میانگین  شتاب معادل کلی بدن در خودرو پراید برابر با 2m/s 62/0به دست آمد که از 2 خودرو دیگر بیشتر بود. میانگین شتاب معادل کلی بدن در تمامی خودروها در ناحیه هشدار سلامتی قرار داشت. میانگین دوز ارتعاش برای تمامی خودروها کمتر از حدود مجاز پیشنهادی استاندارد  (ISO 2631-1 )بود. همچنین افزایش کارکرد ، عمر خودرو و وجود ترافیک در طول رانندگی باعث افزایش مواجهه با ارتعاش در رانندگان تاکسی بود. نتیجه گیری: با توجه به شیفت های کاری نسبتا طولانی رانندگان تاکسی (8 تا 12 ساعت در روز)  و ترافیک سنگین معابر شهری این رانندگان در معرض مواجهه با مقادیر قابل توجه ارتعاش تمام بدن هستند که می تواند باعث اثرات سو بر سلامت آن ها شود. بنابراین باید اقدامات کنترلی فنی-مهندسی و مدیریتی  جهت کاهش اثرات نامطلوب ارتعاش در این افراد ، انجام شود.   این مقاله حاصل پایان نامه کارشناسی ارشد، دانشگاه علوم پزشکی شهید صدوقی یزد می باشد.
